# The classification of walking and phases of gait using EEG: a scoping review

**DOI:** 10.1186/s12984-026-02076-6

**Published:** 2026-06-30

**Authors:** Cormac Ryan, Conor White, Olive Lennon

**Affiliations:** School Public Health, Physiotherapy and Sports Science , University College Dublin, Dublin, Ireland

**Keywords:** Electroencephalography (EEG), Gait phase classification, Neurorehabilitation, Exoskeleton, Brain–computer interface (BCI)

## Abstract

Brain-computer interfaces (BCIs) have the potential to optimise robotic-assisted gait rehabilitation by closing the loop between user and device. Developing such systems requires reliable methods for classifying walking and its component phases to enable real-time feedback. However, research into electroencephalography (EEG)-based gait classification remains limited, with no consensus on optimal methodological or classification approaches. This scoping review aimed to identify (i) gait states and phases classified to date, (ii) methodological processes employed in gait and gait phase classification and (iii) classification systems used. A pre-registered scoping review was conducted. SCOPUS, EMBASE, PubMed and Web of Science were searched. Studies investigating EEG-based gait or gait phase classification, with or without robotic assistance were included. From 15,915 unique studies, 62 were included. Ten studies classified gait phases, with most limited to two phases (*n* = 5). The majority involved treadmill walking (*n* = 8), with only two investigating overground walking. Only one study addressed gait phase classification (swing versus stance) during robotic-assisted gait training (RAGT), achieving a maximum accuracy of 83.06%. No studies investigated gait phase classification in a neurological population. Almost all gait phase studies used offline analysis (*n* = 9). One study performed online analysis, achieving an accuracy of 82.3% for swing versus stance classification. The remaining studies classified gait against other forms of movement. Sensorimotor area electrodes were most frequently used for classification and sensorimotor regions consistently exhibited gait-related discriminative EEG features. Collation of methodological processes identified little consensus within the field. Furthermore, classification accuracy was variable. This scoping review summarises EEG-based gait classification systems, highlighting the need for methodological consensus. Knowledge gaps include the classification of more than two gait phases during free overground walking and limited RAGT-based studies. Notably, gait phase classification research in neurological populations is entirely lacking. Addressing these gaps is critical to advance BCIs for gait rehabilitation.

**Supplementary Information:**

The online version contains supplementary material available at https://doi.org/10.1186/s12984-026-02076-6.

## Introduction

Gait dysfunction is a common complication of central neurological pathology (CNP), with up to 80% of stroke survivors continuing to experience gait impairment in the chronic phase [1]. Regaining the ability to walk independently is consistently highlighted as a key priority for those with neurological conditions [2, 3], with repetitive task practice widely considered an important rehabilitation principle for the restoration of motor function [4]. Despite this, a lack of resources (i.e. staffing, expertise), as well as limited access to consistent and long-term services makes achieving the required levels of rehabilitation intensity and frequency a significant clinical challenge [5].

Robotic-assisted gait training (RAGT) presents a viable option to aid in delivering the increased practice demands in rehabilitation settings [6, 7]. Established advantages of RAGT include the decreased need for human resources to facilitate gait, as well as the ability to deliver high repetitions of walking when compared to therapist-assisted gait [1, 8]. However, there is a need for gait rehabilitation programmes to be better grounded in neurophysiological evidence [9]. Significant cortical involvement is observed when walking in natural overground environments [10, 11]. This works in conjunction with well-established central pattern generators and spinal networks [12]. However, there is insufficient evidence that precise repetition of tasks alone in a robotic device is sufficient to stimulate these pathways and stimulate positive neuroplasticity [13]. The mostly passive nature of movement during RAGT and the inability of RAGT devices to respond to the user’s movement intent and adapt to their preferred movement pattern [14], currently limits their utility in promoting motor problem solving, motor learning and motor skill acquisition.

Human machine interface (HMI) technology, where human information can be used to control an external device, may overcome some of the current limitations of RAGT devices and enhance robotic neurorehabilitation outcomes [15, 16]. Gait and gait phase classification has been successfully achieved using peripheral HMI bio-signals such as electromyography (EMG) [17–19], inertial measurement units (IMU) [20, 21] and foot switches [21], consistently achieving high classification accuracies. However, these methods reflect peripheral neuromuscular and biomechanical activity rather than cortical activity. A specific subset of HMI technology is brain-computer interface technology (BCI) in which cortical activity is the control centre. BCI technology most often employs electroencephalography (EEG) as it is non-invasive, portable, lower in cost than other technologies [12, 16] and in more recent years wireless. Although EEG signals only marginally precede EMG signals [22], an EEG-based BCI robotic gait trainer aligns with the biological, top-down hierarchical organisation of motor control, in which cortical commands precede and regulate peripheral muscular activity [23]. As such, movement classification and feedback based upon EEG activity represent a more natural and physiologically consistent method for responsive rehabilitation robotics that promotes positive neuroplasticity through brain training. In the context of neurological rehabilitation and application of these methods to rehabilitation robotics, EEG-based movement classification offers a means to initiate movement in a manner most closely aligned with how movement is generated in healthy individuals. While peripheral methods such as EMG and IMUs have demonstrated superior classification accuracies [17–21], they do not capture the cortical processes underlying movement generation and therefore cannot provide the same neurophysiological basis for rehabilitation that EEG-based methods offer. EEG-based classification remains in the early stages of development, with the performance gap likely reflecting the greater complexity of classifying cortical signals [24–27]. Early studies demonstrate moderate accuracy in classifying imagined movement, captured using EEG, to control orthoses in a RAGT treadmill device [28] but accuracy classifying actual and overground movement is required to advance the field.

EEG-based gait pattern recognition can be broadly divided into two categories: systems that detect movement intention from pre-movement signals, and systems that classify movement execution from signals generated during movement [29]. Classification of movement intention in both healthy and neurological subjects during normal walking and RAGT has been achieved [30, 31]. However, the classification of movement execution (rather than intention or imagined movement) is essential for real-time gait monitoring and perhaps, most importantly, for providing feedback to the user. Crucially, classification based on motor execution more accurately represents the movement itself and its subcomponents, such as stance and swing phase. Irrespective of approach, EEG-based gait classification has proved challenging [32–34]. Preliminary data provide proof of concept that EEG-based neural networks can accurately classify double stance as well as right and left single-leg stance during free treadmill walking [35], as well as swing and stance phase during both free overground walking and exoskeleton assisted overground walking [36]. However, further studies are required to advance the pool of research using EEG to classify free overground walking (and its phases) as well as during RAGT.

Accurate online gait classification systems using EEG could revolutionise the future of rehabilitation robotics [37]. Firstly, by tailoring the robotic devices to aid the user to a suitable degree and with an appropriate movement pattern and secondly, by progressing towards closing the human machine interface loop [34]. However, the absence of guidance and standardised conventions on how gait is classified and what classification systems are optimal for BCI applications may present a barrier to advancing this field. Therefore, a scoping review of EEG-based gait and gait phase classification systems for motor execution is proposed to collate the current state of the art, including methodologies and applications and to make recommendations for future works. As the broad body of literature addressing gait classification will be examined, a scoping review is a valid approach [38].

The primary aim of this scoping review is to collate and summarise the research field addressing EEG-based classification of gait (and phases of gait). Specific objectives include:

1. To systematically identify and report all studies classifying gait and/or its relevant sub-phases using EEG.
2. To determine how gait is commonly broken down into phases and described in classification studies using EEG.
3. To determine what gait (and/or its relevant phases) is being classified against and with what degrees of accuracy.
4. To describe the processes employed in the identified studies including but not limited to, technology used, data collected, artifact management strategies, feature extraction approaches and classification systems used.
5. To map the cortical activity described in the literature for the phases of gait.

## Methods

A scoping review was conducted, pre-registered with the Open Science Framework [39] and completed in accordance with the Joanna Briggs Institute (JBI) methodology for scoping reviews [40]. A detailed protocol was published prior to the review [41]. Reporting adhered to the PRISMA Extension for Scoping Reviews (PRISMA-ScR) guidelines [42] and a PRISMA-ScR checklist was completed (see supplementary material).

### Population, concept, context (PCC)

The population of interest was defined as healthy adults with no significant past medical history or adults with a CNP. Paediatric populations were excluded. For this review, a CNP was considered as any condition affecting the central nervous system (e.g. stroke and other brain injury, spinal cord injury). Inclusion of both healthy and neurological cohorts allowed the authors to explore the top-down control of gait and whether meaningful cortical activity is retained in those with CNP, as these are the people who will utilise RAGT devices most often. The concept of interest was the classification of the motor execution of gait against other states (e.g. standing or turning) or the classification of gait phases from each other. Studies involving treadmill walking, overground walking or walking using a RAGT device (treadmill-based or overground), conducted in controlled or uncontrolled environments, were eligible for inclusion. Studies classifying imagined movement or movement intention only were ineligible. If classification windows or epochs of EEG data contained any period of motor execution, the study was included. Studies utilising EEG in conjunction with other technologies to complete multimodal analysis (e.g. EMG, kinematics) were also deemed of interest and included. All studies using other brain imaging modalities other than EEG were excluded. Complete inclusion and exclusion criteria are available in Table 1.

**Table 1:** Scoping review inclusion and exclusion criteria

	Inclusion criteria	Exclusion criteria
Population	• Healthy adults (18 years or older) with no other significant past medical history • Adults (18 years or older) with any central neurological pathology	• Non-adult populations
Concept	• The classification of gait against any other state • The classification of the phases of gait • The classification of epochs including any movement execution (even if the intention period is greater than that of the execution period within the same epoch)	• The classification of imagined movement, movement intention only • The classification of upper limb movement • The classification of walking while dual tasking (physical or cognitive) • The classification of gait when one class is a composite of gait and non-gait states (e.g., “walk/relax” vs. another state), as this prevents interpretation of what the classifier is distinguishing • The classification of turning or intention to turn only • The classification of intention to stop or stopping only
Context	• Studies utilising EEG as means of measuring brain activity • Studies utilising EEG in conjunction with other technology to complete multimodal analysis e.g. EMG, kinematics etc	• Studies utilising any other technological means to measure brain activity • Studies using invasive EEG
Types of evidence	• Randomised control trials • Cross-over or quasi randomised control studies • Case control studies • Cohort studies • Cross-sectional studies • Case series and case reports • Conference papers • All publication dates • Full-text articles only • English language only	• Reviews • Opinion pieces • Editorials • Conference abstracts • Protocols • Trial registries

### Search strategy

The search strategy was developed by the primary research team (CR, OL) in conjunction with a liaison librarian. It was guided by the structured tool for the Peer Review of Electronic Literature Search Strategies (PRESS) checklist [43]. Controlled vocabulary terms (e.g., MeSH terms) and appropriate synonyms for controlled vocabulary terms, truncation and different spelling conventions (e.g., “categorisation” versus “categorization”) were used during the search. Search strings related to the key concepts of “EEG”, “gait” and “classification” were developed and combined using Boolean operators e.g. “EEG” AND “gait” AND “classification”. A complete search string is available in the supplementary material. Searches were conducted across SCOPUS, EMBASE, PubMed and Web of Science databases from inception until 10/03/2025. Identified studies were exported to Covidence software for screening and data extraction. Citation management was completed using EndNote software.

### Screening

Identified studies were screened independently by two researchers (CR, CW) against eligibility criteria, firstly by title/abstract and then by full-text review. At each stage, the reviewers’ decisions were recorded, blinded to each other’s decision. Where disagreements arose, they were discussed and reviewed until consensus was reached. A third reviewer (OL) was available to discuss disagreements. The research team contacted the respective authors on two separate occasions when studies contained unclear or missing data. Studies were included despite missing data, provided inclusion criteria were met. No publication date or study methodology restrictions were applied. A log of excluded studies and reasons for exclusion was maintained. No quality appraisal of included studies was conducted, in accordance with the guidelines for scoping reviews [38, 40, 42].

### Data charting, analysis and synthesis

A data charting proforma was developed using Covidence to extract pertinent data from each included study. The template captured a broad range of information, including authors, year of study, country the study was conducted in, participant profiles, experimental conditions, data capture technologies, signal processing strategies, feature selection, classification methods, and model performance metrics. Data were summarised in tabular format and by narrative description, where relevant. Tables were utilised to present key study characteristics and results, while the accompanying narrative text was used to highlight patterns, trends, challenges, and gaps across the literature. Where reported, classification accuracy was defined as the proportion of correctly classified movement epochs relative to the total number of movement epochs. As accuracy was the most frequently reported performance metric across included studies, it was used as the primary basis for comparison. All performance figures are reported as percentage accuracy unless otherwise stated.

## Results

### Summary

**Fig. 1:**
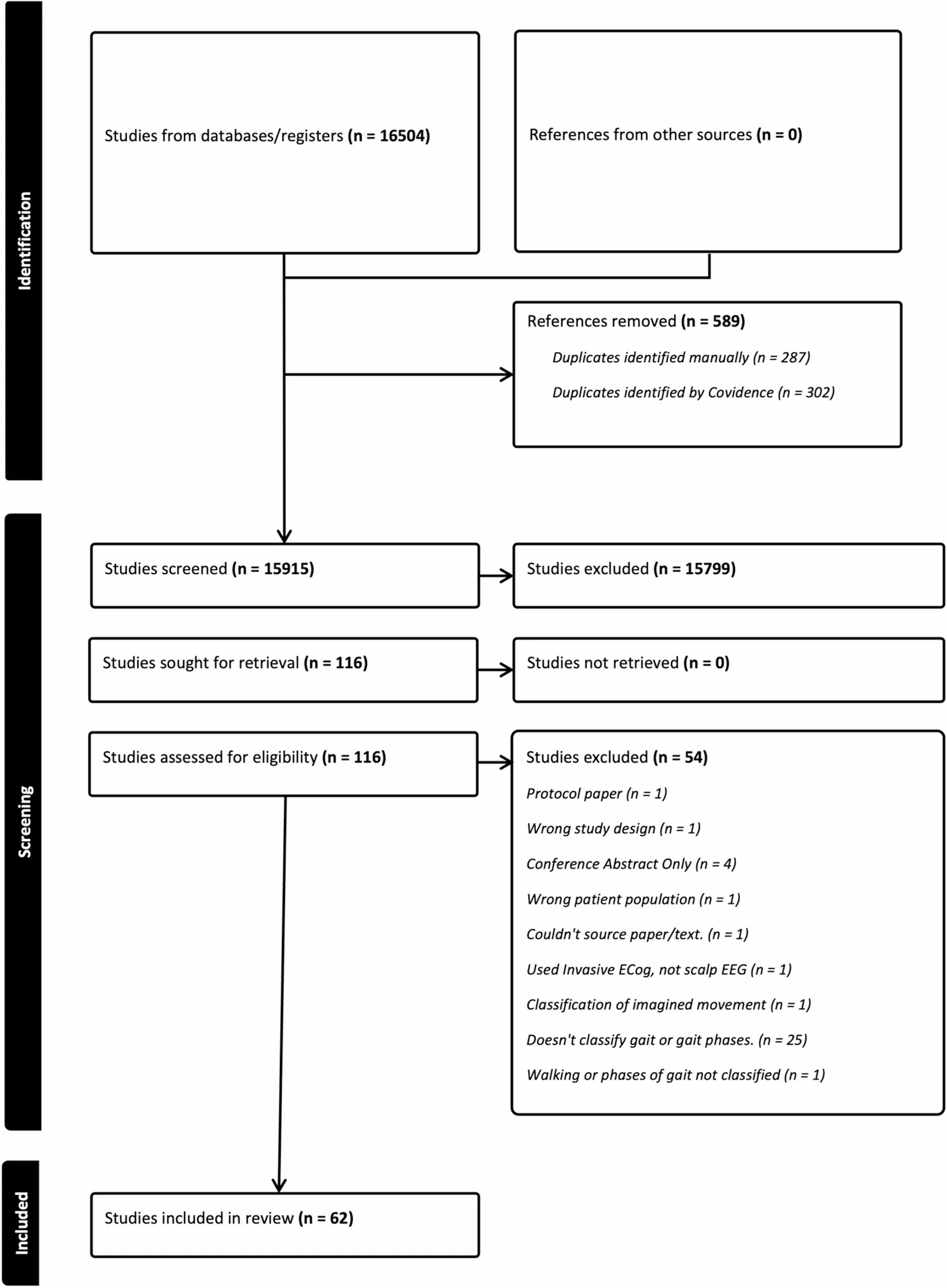
PRISMA flow chart

**Fig. 2:**
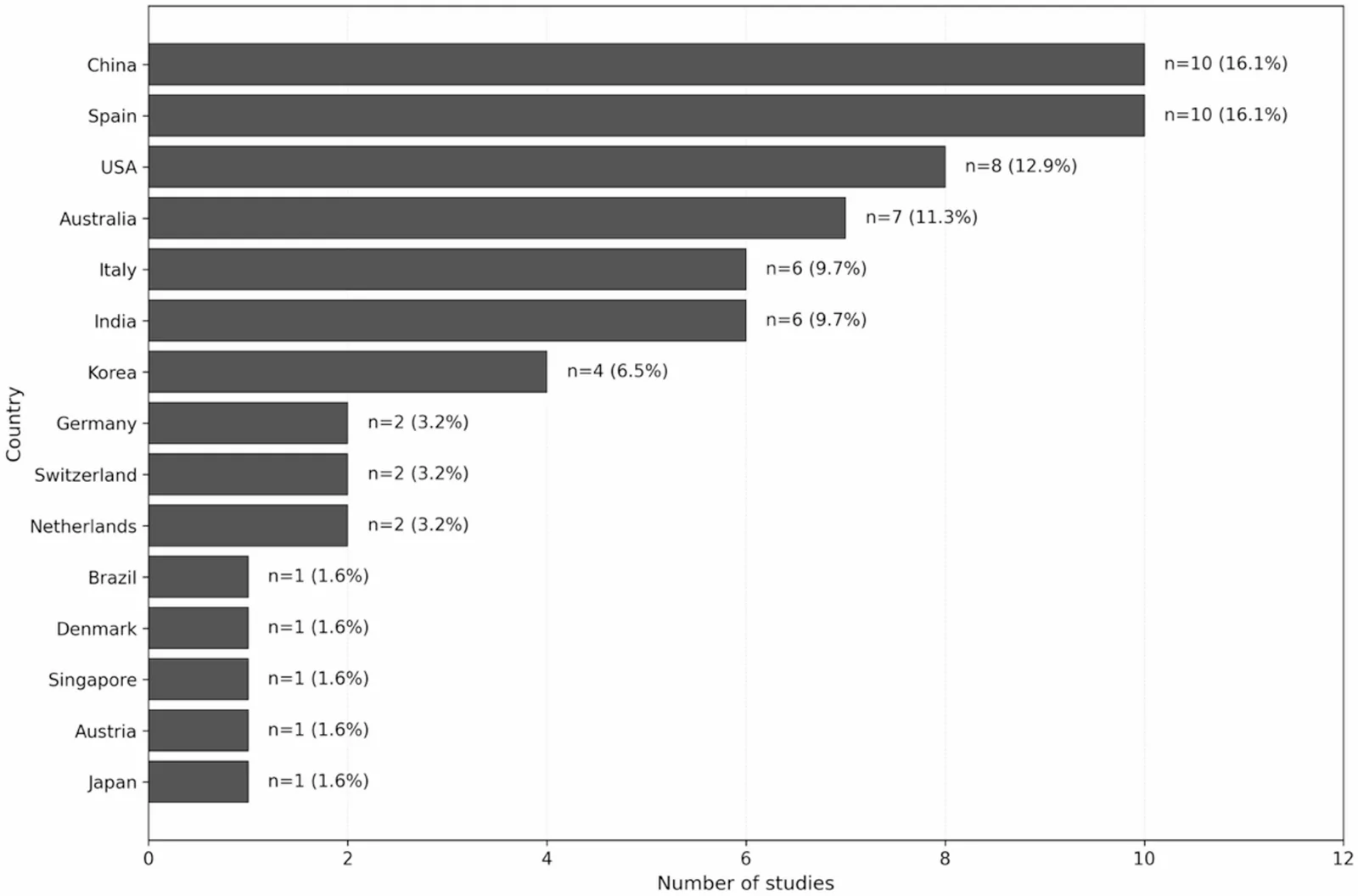
Number of (n = x) and percentage of (%) included studies by publication country

**Fig. 3:**
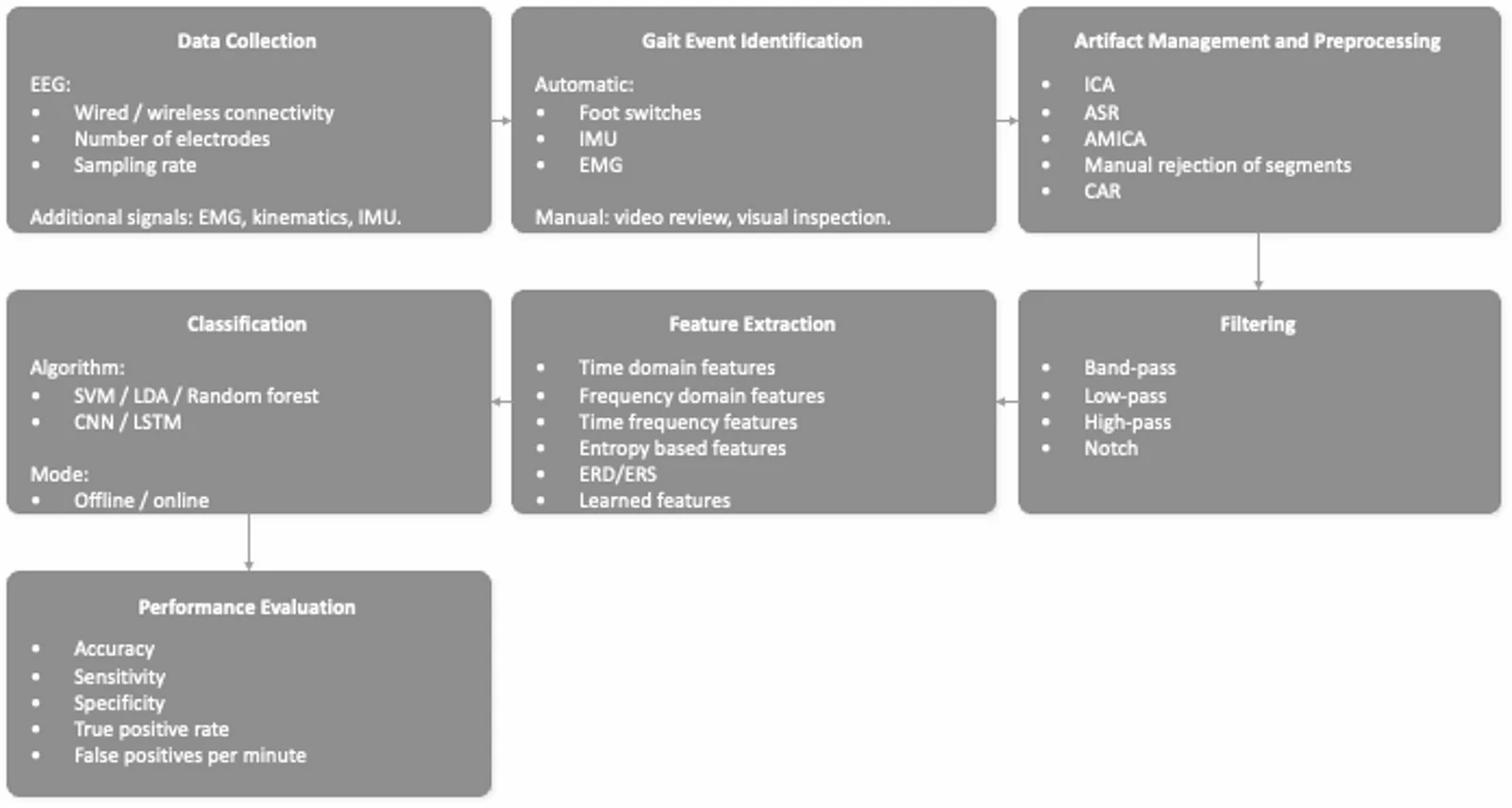
General methodological pipeline of EEG-based gait and gait phase classification studies included in this review

A PRISMA flow chart (Fig. 1) provides a summary of the review process. Of the 15, 915 unique studies identified, 62 met the criteria for inclusion in this scoping review. Included studies were published between 2012 and 2025. The two most common countries of origin for research in this field were China (*n* = 10; 16%) and Spain (*n* = 10; 16%), as detailed in Fig. 2. In total, included studies involved 536 healthy people drawn from 49 studies, 170 people with Parkinson’s disease (PwPD) drawn from 12 studies, 19 people with spinal cord injury (PwSCI) drawn from 5 studies and 11 stroke survivors drawn from 2 studies. The general methodological pipeline identified across included studies is illustrated in Fig. 3. Key approaches identified within each stage are reported under each pipeline heading.

Table 2 details the number of included studies by cohort involved, the classification tasks examined, and walking conditions employed. Across the studies reviewed, sample sizes varied widely, ranging from single-subject designs [44] to larger cohorts of up to 75 participants [45]. Apart from one study which classified gait in an outdoor environment [46], all other studies (*n* = 61) were completed in controlled indoor environments.

**Table 2:** Number of studies per classification task and walking condition, in healthy and central neurological pathology cohorts, with total included subjects detailed as (n = x)

	Walking Overground	Treadmill Walking	Treadmill-Based RAGT	Exoskeleton Assisted Walking
Healthy people				
Gait phases	2 (*n* = 20)	8 (*n* = 56)	**0** (*n* =** 0**)	1 (*n* = 10)
Walk vs. other	30 (*n* = 352)	7 (*n* = 81)	2 (*n* = 20)	7 (*n* = 79)
People with a CNP				
Gait phases	**0** (*n* =** 0**)	**0** (*n* =** 0**)	**0** (*n* =** 0**)	**0** (*n* =** 0**)
Walk vs. other	16 (*n* = 193)	**0** (*n* =** 0**)	1 (*n* = 3)	2 (*n* = 3)

Column totals may exceed the number of included studies as some studies examined more than one walking condition

### Gait phase identification

All ten studies investigating gait phase classification identified individual gait phases by using kinematic data to detect precise temporal markers of key events such as heel-strike and toe-off. As data were collected synchronously, EEG segments were time-locked to these events to create phase-specific EEG classification windows. Papers identified gait events through various means including electrogoniometers (*n* = 1) [47], shank accelerometers and gyroscopes (*n* = 2) [36, 48], motion capture systems (*n* = 4) [49–52], foot switches (*n* = 2) [53, 54] or in one study, a combination of IMU and force plates (*n* = 1) [35].

### Data collection

The majority (*n* = 49; 79%) of included studies collected multimodal data, while the remainder (*n* = 13; 21%) collected EEG data only. EEG in combination with kinematic data was most utilised (*n* = 20; 32%). Wired EEG was used in 31 (50%) studies, while wireless EEG was used in 25 (40%) studies. Six (10%) studies did not report connectivity status, and clarification could not be obtained from the authors upon request. The number of electrodes used across included studies varied from a minimum of 6 electrodes [45, 55–59] to a maximum of 120 electrodes [60]. Most electrode montages followed the international 10–20 electrode placement system (*n* = 38; 61%), 10–10 system (*n* = 12; 19%) or a custom montage (*n* = 7; 11%). Data for 5 (8%) studies could not be obtained. EEG sampling rates ranged from 100 Hz [31, 47, 60, 61] to 2.5 kHz [60].

### Artifact management and preprocessing methods

Use of programmed artifact management techniques, preprocessing strategies and referencing methods was common across included studies. Independent component analysis (ICA) was the most commonly used artifact rejection method (*n* = 24; 38%), followed by artifact subspace reconstruction (ASR) (*n* = 14; 23%) and adaptive mixture independent component analysis (AMICA) (*n* = 5; 8%). Manual rejection of segments and visual inspection/removal were also commonly reported (*n* = 14; 23%). In addition to artifact rejection methods, common average referencing (CAR) was employed as a referencing strategy in ten studies (16%). The majority of included studies also employed data filtering strategies (*n* = 59; 95%). This item was unreported in three (5%) studies.

### Classification tasks and performance results

Considerable heterogeneity was observed across studies in the performance metrics reported. While accuracy was the most frequently reported metric (*n* = 56; 90%), a subset of studies (*n* = 6; 10%), reported alternative or additional metrics including sensitivity, specificity and others only, precluding direct comparison across all included studies.

#### Gait phases

Of the 62 studies included, ten (16%) classified phases of the gait cycle. Eight of these (13%) were completed during treadmill walking. The remaining two studies (3%) involved overground walking. One study described gait phase classification during overground walking using an exoskeleton device [36]. No studies identified classified gait phases in a neurological population under any condition. Table S1 provides a detailed summary of the studies identified classifying gait phases. Discriminating between two phases of gait (swing vs. stance) was the most common classification convention examined (*n* = 5) [36, 47–49, 53]. Two studies reported three class gait phase classification; [35] classified right stance vs. left stance vs. double support and [54] classified right swing vs. left swing vs. double support. Three studies classified seven distinct gait phases, consisting of loading response vs. mid-stance vs. terminal stance vs. pre-swing vs. initial swing vs. mid-swing vs. terminal swing [50–52].

#### Gait phase classification accuracy

As detailed in Table S1, gait phase classification accuracy in studies involving treadmill walking (*n* = 8) ranged from 44% [54] to 98.56% [51]. Gait phase classification accuracy in studies employing overground walking ranged from 40% [48] to 89.32% [36]. Gait phase classification accuracy during RAGT ranged from 82% to 83.06% depending on exoskeleton mode employed in one study [36]. With respect to the classification of two gait phases (swing vs. stance), accuracies ranged from 40% [48] to 93.71% [49]. For three-class classification, reported performance ranged from 83% overall accuracy [35] to class-wise accuracies of up to 97% [54]. Classification accuracies ranged from 71.9% [52] to 98.56% [51] for seven gait phases. The one study addressing gait phase classification in an exoskeleton [36] achieved a peak accuracy of 83.06% for the classification of swing vs. stance phase with an exoskeleton in transparent mode.

Two studies directly compared EEG-only-based gait phase classification with approaches incorporating EMG signals [51]. reported that classification accuracy for seven gait phases decreased from 98.56% using a combined EEG-EMG system to 68.36% when using EEG alone. Similarly, [54] evaluated EEG-based classifiers paired with varying amplitudes of EMG for the classification of right swing, left swing, and double support phases. When EMG amplitude within the classification model was reduced from 100% to 10%, classification accuracy decreased from 93% to 80% for double support, from 97% to 74% for right swing, and from 96% to 44% for left swing. All gait phase classification studies conducted offline analyses, reporting accuracies ranging from 40% [48] to 98.56% [51] across studies classifying different numbers of gait phases. One study conducted online classification [53], achieving an accuracy of 82.3% for swing versus stance phase classification during RAGT.

#### Classification of walking against another state

The majority of papers (*n* = 52; 84%) classified walking against other states. Over half of these studies (*n* = 33; 53%) involved healthy subjects only, while 13 studies (21%) involved subjects with CNP only. A small number of studies included both healthy and CNP participants (*n* = 6; 10%). The majority of studies classifying gait versus other states conducted their classification offline (*n* = 37; 71%). A smaller proportion conducted pseudo-online classification (*n* = 8; 15%) or online classification (*n* = 12; 23%). To enable synthesis across studies, non-walking comparators were grouped into the following categories: (i) static or resting states (*n* = 15; 24%), (ii) other walking or locomotor tasks (*n* = 36; 58%), and (iii) pathological or altered gait states (*n* = 13; 21%). A small group of studies (*n* = 9; 15%) classified RAGT-based walking versus other RAGT states, with these reported separately below. As many studies classified walking against several states, studies were counted in all relevant comparator categories.

#### Classification accuracy of walking against another state

Table 3 summarises the reported performance ranges for classification of walking versus respective comparator groups under offline, pseudo-online and online evaluation modes. The classification of walking against static or resting states displayed the best performances in both offline and online modes, achieving maximum performances of 100% and 85.6% respectively. The classification of walking against other walking or locomotor tasks achieved maximum performances of 80.3% to 96.7% across evaluation modes. The classification of walking against pathological or altered gait states was only trialled in offline modes. All studies describing the classification of pathological gait states involved PwPD, achieving classification ranges from 55.6 to 95.8% for freezing of gait (FOG) detection. The classification of walking against altered gait states involved only one study [62], describing the classification of normal walking versus an anticipated fall, achieving an accuracy of 85%. With respect to those studies which classified RAGT-based walking versus other RAGT states, offline accuracies ranged from 60.34% [63] to 98% [60], pseudo-online true positive rates ranged from 42% [64] to 86% [65], while online true positive rates ranged from 33% [64] to 100% [66, 67]. All studies describing the classification of walking versus other states are detailed in Table S2.

**Table 3:** Accuracy ranges for the classification of walking vs. other states

Offline		
Walking vs.	Minimum accuracy	Maximum accuracy
Static or resting states	69% [46]	100% [68]
Other walking or locomotor tasks	20% [69]	96.7% [70]
Pathological or altered gait states	55.6% [71]	95.8% [72]

Pseudo-online		
Walking vs.	Minimum TP rate	Maximum TP rate
Static or resting states	31.7% [73]	68.7% [73]
Other walking or locomotor tasks	40.2% [74]	93.14% [75]
Pathological or altered gait states	N/A	N/A

Online		
Walking vs.	Minimum TP rate	Maximum TP rate
Static or resting states	70% [76]	85.6% [77]
Other walking or locomotor tasks	33% [64]	80.3% [74]
Pathological or altered gait states	N/A	N/A

Accuracy (%) is reported for offline classification. TP denotes true positive detection rate (% of gait events detected within the predefined temporal tolerance). N/A indicates metric not applicable

### Classification methods

#### Features for classification

Traditional hand-crafted features including time-domain, frequency-domain, time-frequency, ERD/ERS (event-related desynchronisation/event-related synchronisation) and entropy-based features were most commonly used across studies (*n* = 45; 72%). Feature projection methods such as PCA and ICA were used less frequently (*n* = 6; 10%), while automatic feature learning approaches (including deep learning models trained on raw EEG signals), were reported in five studies (8%). Six studies (10%) utilised a combination of these feature categories. Detailed feature selections for individual studies are detailed in Table S1 and S2.

#### Classification models

Traditional machine learning was most commonly used (*n* = 40; 65%), with SVM and related variants reported in 26 studies (42%), followed by LDA (*n* = 10; 16%) and RF (*n* = 8; 13%). Deep learning models were also prevalent (*n* = 22; 35%), with CNN-based models (*n* = 15; 24%) and LSTM/RNN-based models (*n* = 11;18%) most common. Many studies evaluated more than one model (*n* = 25; 40%). Table S1 and S2 detail the classification models employed in included studies and respective accuracies.

### Cortical activity

Over a quarter of included studies (*n* = 16; 26%) mapped cortical activity related to gait. Across these studies, sensorimotor regions, including fronto-central and central midline areas, were most frequently identified and were consistently reported to exhibit gait-related modulation and discriminative EEG features associated with gait phases [31, 35, 37, 65, 76, 78–80]. Parietal cortical involvement was also reported in several studies, particularly in studies involving gait adaptation or changes in walking conditions [36, 45, 74, 81–83]. Lastly, increased frontal cortex activity was reported in studies investigating gait adaptation, exoskeleton-assisted walking, or pathological gait states such as freezing of gait [31, 36, 57, 82, 83].

## Discussion

This scoping review synthesises the current literature on EEG-based gait and gait phase classification, highlighting significant methodological heterogeneity and limited clinical translation. While some common trends are identified such as a focus on sensorimotor area signal acquisition sites and a broad consensus on the importance of artifact rejection/correction and filtering strategies, considerable divergence is noted in filtering methods used, feature selection and classification algorithms employed. Such heterogeneity presents a fundamental challenge in drawing firm conclusions and determining the feasibility of EEG-based gait phase classification for clinical applications. This scoping review maps the current state of the art while outlining critical steps required to advance the field towards clinically viable, closed-loop BCI systems for RAGT.

### Gait phase classification

There is a paucity of studies addressing gait phase classification, particularly during overground walking and RAGT, with the majority of included studies addressing treadmill walking. The classification of two gait phases, mainly swing and stance, was the most common task investigated, with performance results suggesting that this remains a challenge. It is worth noting that the classification of swing versus stance phase was the only classification task attempted during free overground walking or during RAGT, potentially impacting classification performance. However, such tasks will be required for future clinical translations. Despite promising results in some studies classifying swing versus stance phases of gait, the binary approach is unlikely to be sufficiently detailed for clinical applications.

The double support phase which constitutes 20%–25% of the gait cycle in healthy individuals [84] and is often prolonged in neurological populations [85, 86], is largely under-investigated. This phase is clinically significant for balance and weight transfer [86] not only in healthy overground walking but also in RAGT in neurological populations. The double support phase and the weight transfer which occurs during it is functionally critical for propulsion generation and step initiation in exoskeleton assisted walking [87, 88]. Furthermore, RAGT studies have shown some initial promise in improving temporal characteristics of gait, including a reduction in double support time [86, 88], as well as functional status [88, 89]. Despite the clear importance of the double support phase, it was rarely addressed in the included studies, with only two studies attempting classification of three gait phases (i.e. swing phase, stance phase and double support phase), both limited to treadmill-based walking conditions [35, 54]. Given the significant proportion of the gait cycle constituted by the double support phase, its role in weight transfer, balance and step initiation and its increased duration in neurological populations, it is imperative that future classification studies consider it more often, particularly if RAGT devices are to become more responsive to the user and deliver phase-specific assistance. Although there is evidence of increased EEG power and synchronisation during periods of double support [12, 90], which theoretically may allow for easier classification, in practice this did not always translate in the few papers that considered it [36]. One study [54] which did achieve promising initial results when classifying three gait phases, including the double support phase, used a combined EEG–EMG system. However, classification accuracies dropped significantly when the EMG amplitude fed into the system was reduced to 10%. This suggests that EEG alone may be insufficient for reliable double support classification at present, further highlighting the need for ongoing research in this area. While the classification of seven distinct gait phases across three included studies [50–52] represents a novel achievement, the clinical utility of such granularity for exoskeleton control remains unclear. It should also be noted that these studies were treadmill-based and used lower-than-normal gait speeds, which likely presented more uniform data with less artifact. Furthermore, similarly to [54,51] also displayed reduced classification performance with the removal of EMG from the classification model. A further challenge is highlighted by [51], who identified that while multi-class classification of seven gait phases is feasible, it is computationally costly for online use, an important consideration for clinical applications.

Despite evidence of phase-dependent neural signatures during gait [10, 12, 91], which may provide discriminative power [92], the limited spatial resolution over lower-limb sensorimotor areas clearly limits hemispheric differentiation, challenging gait phase classification [54, 93]. Therefore, there is a need for caution in explicitly linking scalp-measured EEG activity to gait phases, as brain signals reflect not only muscular activity but also sensation, planning, integration and coordination [10]. Moreover, previous works have concluded that substantial disagreement remains regarding the cortical correlates of gait in people who have experienced a stroke [33, 94]. Further research is required to build repositories of neurophysiological gait phase data that can then be used as reference data for future studies. Currently, no consensus exists about what constitutes sufficient gait phase classification for closed-loop BCI technology, specifically how many phases may be necessary.

The challenge of gait phase classification is often accentuated further during RAGT, due to increased artifact, induced by the presence of robotics [95]. This is exemplified in the findings of this scoping review, with only one study attempting gait phase classification in a RAGT device [36]. Furthermore, no study to date has attempted gait phase classification in a CNP population. This is likely due in part to the highly individual pathophysiology and presentations of individuals with CNP. Overground, walking in neurological cohorts has been shown to be inconsistent and less automatic [96], while a recent systematic review highlighted a lack of consensus with respect to the cortical changes that occur in CNP populations during RAGT [94]. This is particularly problematic given that individuals with CNP are the primary users of RAGT devices and may benefit most from advances in BCI engineering. Therefore, it is essential that future studies focus on EEG-based classification within these cohorts under RAGT conditions to better inform device design and optimisation.

### Classification of walking against other states

This scoping review highlights that the classification of walking against other states using EEG is more commonly researched, with reasonable success reported. However, some specific areas of research were lacking, with most studies identified including healthy individuals and few studies considering CNP populations. Furthermore, most studies involved free overground walking, with only a limited pool of papers (*n* = 9) classifying walking against another state during RAGT. Only a third of these RAGT studies include participants with CNP. Our findings are similar to several comparable systematic reviews within the broader research field in which neurological cohorts were also under-researched in both normal walking and RAGT conditions [27, 33, 94, 97]. This pattern suggests that the underrepresentation of neurological cohorts is a persistent and systematic gap in the field, one that must be addressed if EEG-based classification is to advance towards clinical applications. This is especially true given that such RAGT devices are designed to be used with neurological cohorts.

Results of this scoping review also reflect substantial heterogeneity with respect to the classification comparator tasks utilised, limiting the ability to draw firm conclusions about the effectiveness of EEG-based classification across studies. As expected, classification of walking versus static or resting states generally achieved higher accuracies than classifications involving other comparator groups. This likely reflects the substantial differences between resting state EEG and gait related cortical activity, which may be more readily distinguishable by classification algorithms, consistent with evidence of greater cortical activation during mobility versus rest [33, 98]. However, the substantial inter-individual variability in the spatial and spectral patterns of this activity, particularly in CNP populations [33, 94], cautions against assuming a uniform cortical profile and presents a challenge for its reliable use in gait classification. This highlights the need to develop repositories of neurological cohort data and suggests that subject-specific EEG-based gait classification may be required if it is to be reliably applied in clinical settings.

With respect to classification of walking versus other walking or locomotor states, variability in classification accuracy was observed, indicating a greater classification challenge, likely due to the similar nature of the movement classes. Evidence suggests these activities elicit modulations in overlapping cortical regions, producing similar neural patterns rather than distinct states [12]. Notably, studies classifying walking against pathological or altered states, including those involving CNP populations, do not show reduced classification accuracies compared to other comparator groups. As the majority of these studies involved PwPD, this may reflect the broadly increased cortical activity, including compensatory recruitment of sensorimotor and prefrontal regions [99]. Such neural adaptations may facilitate differentiation by classification algorithms, suggesting that future research should prioritise neurological cohorts to better reflect the clinical populations for whom these systems are ultimately intended.

### Cortical activity

Despite only a quarter of studies reporting cortical activity mapping, there is broad consensus on the dominance of the sensorimotor cortex in gait production and control, consistent with well-established models of locomotor control in which primary, premotor and supplementary motor areas are heavily involved [12, 100]. Critically, wider evidence of task and phase-specific modulation during overground and treadmill walking [10, 91], as well as increased frontal cortex activity during RAGT [94] supports the potential of these areas as meaningful biosignatures for gait phase classification. The implication that higher order executive processes are involved when automatic locomotor control is challenged strengthens the neurophysiological rationale for EEG-based control of RAGT devices. This represents an important consideration for classification models as the most meaningful neural biosignatures may differ between free walking and RAGT conditions. Furthermore, at present, insufficient evidence is available to draw similar conclusions in CNP cohorts [33, 94, 99] despite some initial findings in this scoping review to suggest the same [31]. Further research is required to establish the effects of RAGT-based walking on cortical activity in neurological cohorts to better inform the design of classification models and EEG-based BCI controlled RAGT devices intended for clinical use.

### Contextualising classification performance

Due to significant methodological and experimental differences across included studies, gait phase classification accuracies must be interpreted carefully. As outlined in the *cortical activity* section of this paper, the neural bio-signals underlying gait differ meaningfully across walking conditions [90, 101, 102], meaning accuracies achieved under treadmill conditions may not generalise to overground walking or RAGT conditions. This is illustrated by the reduced classification accuracies reported in [48], the only gait phase classification study conducted during free overground walking. A similar consideration must be extended to future gait phase classification studies during RAGT due to the increased frontal cortex activity it induces [31, 36]. Furthermore, the data used for classification must be considered when assessing reported accuracies. The superior classification performance observed in multimodal studies hinders comparison with EEG-only approaches. A final consideration is the experimental conditions of the study. At present, ecological validity is limited by the predominance of controlled laboratory environments with only one study [46] attempting, achieving a classification accuracy of 69%; likely reflecting the greater classification challenge due to additional environmental stimuli and increased potential for artifact. Collectively, findings of this scoping review imply that the true clinical utility of EEG-based gait phase classification remains to be assessed in more ecologically representative settings.

### Clinical implications

Current evidence and expert clinical consensus support the use of RAGT as an adjunct to conventional rehabilitation for individuals with chronic neurological conditions [8, 103], with evidence suggesting some learning effects during RAGT [31, 104]. However, it is unclear whether these changes lead to measurable improvements in gait performance or overall function. While studies included in this review represent an advancement towards BCI control of RAGT devices, current classification accuracies are insufficiently robust and inadequately validated in real-time conditions to warrant clinical application at present. Even identified studies achieving higher gait phase classification accuracies are still limited by the presence of false positives that pose potential safety issues for the user in higher risk applications like RAGT, where unwanted parameter selection or movement applied to the user may result in falls [79]. Progress towards clinical translation therefore requires not only improved classification accuracy but rigorous real-time and ecological validation, specifically in neurological populations during both normal overground walking and RAGT.

### Future directions

#### Classification of motor execution of gait phases

The majority of EEG-driven closed-loop BCI systems for RAGT control have relied on motor imagery [24, 26, 105] rather than motor execution. To the best of the authors’ knowledge, the development of a closed-loop overground exoskeleton control system using an EEG-based BCI that utilises online classification of movement execution remains unreported. Future research should focus on characterising EEG correlates of the motor execution of gait phases, so that a neural reference for normal and impaired walking can be established. As discussed previously, this gait phase classification should prioritise the inclusion of double support as a discrete gait phase given its clinical relevance and its role in RAGT step initiation. Such knowledge is fundamental for developing biologically grounded, EEG-driven control systems, thereby restoring a natural, hierarchical sequence of motor control.

#### Artifact

This scoping review highlights that artifact rejection and correction methods, along with filtering techniques are widely used in the field. However, selection of the techniques employed appears to be an arbitrary practice. Furthermore, manual removal of electrodes and/or artifact was often employed, with many electrodes removed based on subjective analysis of electrode signal viability, rather than removal based on established thresholds. This finding is in line with findings from another review of EEG for classification tasks [106], whereby more than a quarter of studies removed artifact manually, a practice that is not suitable for real-time closed-loop BCI technology. The arbitrary and individual nature of current artifact rejection/correction methods highlights a need for agreed and standardised automated artifact management solutions, particularly with a view to future real-life and real-time applications [25, 107].

#### Features for classification

The considerable heterogeneity in features used across included studies reflects the absence of a standardised methodology for EEG-based gait and gait phase classification. Time-frequency features such as ERD/ERS, MRCP, wavelet-derived features and spectral power measures were among the most widely used, suggesting alignment with the well-established role of oscillatory cortical dynamics during movement preparation and execution [10, 91, 94, 100, 104]. Frequency and time-domain features, such as power spectral density (PSD), slope sign change (SSC) and mean absolute value were also frequently employed. The use of these time-frequency, frequency domain and time domain features likely reflects their computational efficiency and interpretability allowing for quicker processing times; key requirements for real-time applications. This trade-off between feature complexity and processing times is evident in [51] whereby higher-dimensional feature sets achieved improved classification accuracies, processing times were as long as 26 s while simpler two-dimensional feature sets displayed classification times of 0.33 s. A similar pattern was exhibited in a further complex gait phase classification study [52] whereby the use of simple SSC features proved effective. Notably, a small number of more recently published studies included in this review (all published in 2023 or later) employed automatic feature learning through deep learning models [47, 67, 71, 72, 108], displaying competitive classification performances. This displays a potentially emerging shift towards automatic feature selection methods. Overall, significant variability exists in feature selection across included studies, limiting comparability. This represents a methodological gap in the field of research. Future works should further explore the potential of automatic feature learning to establish its performance compared to the commonly used hand-crafted feature approaches, with a view to the development of standardised feature extraction protocols.

#### Classification models and mode

The trade-off between classification model accuracy and real-time feasibility remains a core challenge for gait phase classification and BCI applications in assistive devices [109]. A need for increased classification accuracy has led to deep learning models becoming increasingly prevalent in the field, however, they often require longer offline processing, thus limiting real-time application. In contrast, simpler traditional machine learning algorithms like LDA and SVM offer faster processing times, often with only modest reductions in accuracy, and remain the most commonly used in the field. A similar pattern was also noted in a comparable systematic review [110]. This trade-off highlights the need for optimised pipelines and more efficient algorithms that maintain robust classification performance while supporting the responsiveness required for BCI use. Furthermore, similar to previous findings [111], the majority of studies included in this scoping review employed offline classification. Future developments must also focus on advancing from offline to online classification systems to allow for real-world BCI use [112] and application to RAGT devices. However, until online classification systems demonstrate sufficient performance, their integration into clinical RAGT will remain limited.

#### Performance metrics

The lack of standardised performance reporting metrics across studies represents a notable methodological gap in the field. Establishing consensus on evaluation metrics would facilitate more meaningful comparisons and support the development of benchmarks for future research. Until then, the field of research cannot meaningfully quantify progress or evaluate the effectiveness of individual classification systems with the goal of clinical translation.

#### Establishing a unified framework

As outlined throughout this scoping review, despite some areas of consensus, significant methodological heterogeneity exists across the research field including classification tasks, neural bio-signal capturing technology, experimental conditions, electrode configurations, sampling rates, filtering methods, artifact management methods, feature selection, classification algorithms and performance metrics. Such is the degree of variance in classification approaches that the field of research is not yet developed sufficiently to the stage at which a unified methodological framework can be prescribed. Rather than working towards the establishment of best practice guidelines, a more useful approach may be to develop an evaluative framework capable of assessing the contribution of individual methodological approaches to classification performance. Obtaining clarity on the contribution of individual methodological components during gait and gait phase classification would prove an important step towards the development of such a framework.

## Limitations

Some limitations are acknowledged in this scoping review. Firstly, for pragmatic reasons, included studies were limited to the English language only. Therefore, it is possible that studies published in other languages could further inform the area. Secondly, this review applied strict inclusion criteria, considering only studies that classified motor execution, i.e. at least one class contained motor execution within its classification window, thereby excluding some literature focused solely on motor intent. Additionally, the extent of methodological heterogeneity across included studies precluded direct comparison of findings across studies and limited the generalisability of conclusions drawn. Lastly, the predominance of healthy participant cohorts undoubtedly limits the applicability of findings to the neurological populations for whom RAGT devices are primarily intended.

## Conclusions

EEG-based gait and gait phase classification remains a nascent but promising field, characterised by substantial methodological heterogeneity across experimental paradigms, preprocessing pipelines and classification models. EEG-based classification of walking against other states, under both free walking and RAGT conditions, is well-researched, feasible and extends to both healthy and CNP cohorts. However, EEG-based gait phase classification is considerably less developed. Initial findings offer promise for swing versus stance phase classification, however the clinical utility of this approach is limited without the inclusion of the double support phase. Knowledge gaps remain including the classification of more than two gait phases during free overground walking and during RAGT, particularly in CNP cohorts. Before a unified methodological framework can be established, the field would benefit from an evaluative approach to systematically assess the contribution of individual methodological components to classification performance. Addressing these gaps is essential if EEG-based gait phase classification is to be used as a foundation for closed-loop BCI control of RAGT devices.

## Supplementary Information

Below is the link to the electronic supplementary material.

Supplementary Material 1.

## Data Availability

As this article is a scoping review, no new data are generated. All data generated is from previously published research. This scoping review has been registered with the Open Science Framework since 9 March 2025.*Extended data*Extended data related to this scoping review includes the search string utilised as well as a copy of the PRISMA-ScR checklist. These are available as extended data at the following repository.Repository name: *Open Science Framework (OSF).* Title: *‘The classification of walking and phases of gait using EEG: a scoping review’. Registration https://doi.org/ [https://doi.org/10.17605/OSF.IO/WUP6M] . [Ryan] (https://f1000research.com/articles/14-549) [*et al*] (https://f1000research.com/articles/14-549) [. (2025b)] (https://f1000research.com/articles/14-549). Data are available under the terms of the Creative Commons Attribution 4.0 International license (CC-BY 4.0).

## Abbreviations

AMICA: Adaptive mixture independent component analysis
ASR: Artifact subtract reconstruction
BCI: Brain-computer interface
CAR: Common average referencing
CNN: Convolutional neural network
CNP: Central neurological pathology
CSP: Common spatial pattern
DMD-ACSP: Dynamic mode decomposition-adaptive common spatial pattern
DS: Double support
EEG: Electroencephalography
EMG: Electromyography
EOG: Electrooculography
ERD: Event-related desynchronisation
ERS: Event-related synchronisation
FOG: Freezing of gait
FP: False positive
FP/Min: False positives per minute
HMI: Human machine interface
ICA: Independent component analysis
IMU: Inertial measurement unit
JBI: Joanna Briggs Institute
KNN: K-nearest neighbour
L: Left
LDA: Linear discriminant analysis
LSTM: Long short-term memory
LSTM RNN: Long short-term memory recurrent neural network
MDM: Minimum distance to mean
MLPNN: Multilayer perceptron neural network
MPF: Mean power frequency
MRCP: Movement-related cortical potential
N/A: Not applicable
NN1: Neural network 1
NN2: Neural network 2
NR: Not reported
PCA: Principal component analysis
PCC: Population, concept, context
PRESS: Peer review of electronic literature search strategies
PRISMA-ScR: PRISMA Extension for Scoping Reviews
PSD: Power spectral density
PwPD: People with f's Disease
PwSCI: People with spinal cord injury
R: Right
RAGT: Robotic-assisted gait training
RELICA: Reliable independent component analysis
RF: Random forest
SSC: Slope sign change
SSRL: Spatio-spectral representation learning
SVM: Support vector machine
TP: True positive
TP/R: True positive rate
