# Supplementary material for: The classification of walking and phases of gait using EEG: a scoping review
Source: J Neuroeng Rehabil. 2026 Jun 30;23:274. doi: 10.1186/s12984-026-02076-6 (PMC13587400; doi:10.1186/s12984-026-02076-6)
Supplement: Supplementary file 1 [file 12984_2026_2076_MOESM1_ESM.docx]

***Supplementary Material***

**Appendix 1: Table S1: Summary of papers classifying gait phases during unassisted walking and RAGT.**

| **Gait Phase Classification** | | | | | | | | | | |
| --- | --- | --- | --- | --- | --- | --- | --- | --- | --- | --- |
| **Author,**  **Year (Country)** | **Participants**  **(n=x)** | **EEG Device & Connectivity** | **Number of Electrodes & EEG Sampling Rate** | **Additional Data Collected** | **Classification Task**  **(Walking Condition)** | **Artifact Management and Referencing**  **Methods** | **Filtering Applied** | **Features Used** | **Classifier Used**  **(Mode)** | **Performance** |
| ***Unassisted Walking*** | | | | | | | | | | |
| **Blanco-Diaz,**  **2024* [47]**  (Brazil) | Healthy  (n=5) | actiCAP    Wired | 60    100Hz | Kinematics | Swing vs stance.  (Treadmill) | CAR | Band-pass low-frequency (0.1–4 Hz) | Learned features | Convolutional Neural Network (CNN)  (Offline) | 69% |
| **Bodda, 2020* [48]**  (India) | Healthy  (n=10) | EMOTIV    Wireless | 14    128Hz | Kinematics | Swing vs stance.  (Free overground) | CAR | Band-pass EEG (5–60 Hz) + notch | Time–frequency features (temporal & spectral; cortical potentials) | Support Vector Machine (SVM)    Decision trees J48.    Naïve Bayes  (Offline) | **SVM:** 40%    **Decision tree:**60%    **Naive Bayes:** 70% |
| **Herbert,**  **2020 [35]**  (Germany) | Healthy  (n=3) | actiCAP    Wired | 32    1000Hz | Kinematics | Right (R) stance vs left (L) stance  vs double support (DS)  (Treadmill) | ICA | Broadband (0.1–100 Hz) + notch | Time-domain features | Long short-term memory (LSTM)  (Offline) | 83.7% |
| **Jorquera,**  **2013* [49]**  (Switzerland) | Healthy  (n=5) | SynAmps2    Wired | 60    500Hz | Kinematics | Swing vs stance.  (Treadmill) | Not reported (NR) | Band-pass low-frequency (0.1–2 Hz) | Time-domain features (windowed EEG) | Linear discriminant analysis (LDA)  (Offline) | **Individual best electrodes:** 93.71%    **Common set of electrodes:**89.42% |
| **Tortora,**  **2020 [53]**  (Italy) | Healthy  (n=11) | actiCAP    Wired | 64    2048Hz | Kinematics | Swing vs stance.  (Treadmill) | ICA based methods  ASR  CAR | Band-pass (1–98 Hz / 1.5–48 Hz) + notch | Time–frequency features including motor related cortical potentials (MRCP), event related desynchronisation (ERD) and event related synchronisation (ERS). | Long short-term memory recurrent neural network (LSTM RNN)  (Offline, online) | **Offline**  Both legs: 91.2%  R leg: 87.8%  L leg: 88.2%    **Online**  Both legs: 82.3%  R Leg: 80.5%  L Leg: 75.3% |
| **Tortora,**  **2020 [54]**  (Italy) | Healthy  (n=11) | actiCAP    Wired | 64    2048Hz | EMG  Kinematics | R swing vs L swing  vs DS.  (Treadmill) | ICA based methods  ASR  CAR | Band-pass (1–98 Hz / 1.5–48 Hz) + notch | Time-domain features | LSTM  (Offline) | **EEG & EMG**  R swing: 97%  L swing: 96%  DS: 93%    **EEG & 10% amplitude EMG**  R swing: 74%  L swing: 44%  DS: 80% |
| **Wei,**  **2020* [50]**  (China) | Healthy  (n=7) | LiveAmp    Wireless | 24    500Hz | EMG  Kinematics | Loading response vs mid-stance vs  terminal stance vs pre-swing vs initial swing vs mid-swing vs terminal swing.  (Treadmill) | ICA | Band-pass (0.5–50 Hz) + notch | Time-domain features; slope sign changes (SSC) | Support vector machine library  (Offline) | 1.4 km/h: 95.58%  2.0 km/h: 97.63%  2.6 km/h: 98.10% |
| **Wei,**  **2021 [51]**  (China) | Healthy  (n=7) | Custom device    Wired | 24    500Hz | EMG  Kinematics | Loading response vs mid-stance vs  terminal stance vs pre-swing vs initial swing vs mid-swing vs terminal swing.  (Treadmill) | ICA | Band-pass (0.5–50 Hz) + notch | Time-domain features; mean absolute value and SSC. | Grid Search Kernel SVM  (Offline) | ***EEG & EMG:*** 98.56%    ***EEG Only*:**  68.36% |
| **Wei,**  **2021 [52]**  (China) | Healthy  (n=7) | Custom device    Wired | 24    500Hz | EMG  Kinematics | Loading response vs mid-stance vs  terminal stance vs pre-swing vs initial swing vs mid-swing vs terminal swing.  (Treadmill) | ICA | Band-pass (0.5–50 Hz) | Frequency-domain features; mean power frequency (MPF) | SVM Particle Swarm Optimisation  (Offline) | ***SSC EEG & EMG***  @ 1.4 km/h: 95.58%  @ 2.0 km/h: 97.63%  @ 2.6 km/h: 98.10%    ***MPF EEG & EMG***  @ 1.4 km/h: 71.90%  @ 2.0 km/h: 80.63%  @ 2.6 km/h: 87.89% |
| ***RAGT*** | | | | | | | | | | |
| **Tortora,**  **2023 [36]**  (Italy) | Healthy  (n=7) | g.Nautilus PRO    Wireless | 64    250Hz | EMG  Kinematics | Swing vs stance.    (Free overground)    (Exoskeleton overground) | ICA-based artifact rejection with ASR | Band-pass (1–45 Hz) + notch + common average filtering (CAF) | Time–frequency features (event related spectral perturbation) | CNN LSTM  (Offline) | ***Trained from same condition***  Free Walking: 89.32%  Transparent Mode: 83.06%  Adaptive Mode: 82%  Full Mode: 82.14%    ***Trained from free walking***  Adaptive Mode: 58.8%  Full Mode: 60% |

**Footnotes:** * denotes conference paper**.** Filtering information is summarized. Exact cutoff frequencies and filter parameters vary across studies and are reported in the original publications. Feature descriptions are grouped into broad methodological categories (e.g., time-domain, frequency-domain, time–frequency, spatial, connectivity, nonlinear, and learned features) to improve clarity and comparability across studies. Performance is reported as percentage accuracy unless otherwise stated.

**Appendix 2: Table S2: Summary of papers classifying gait vs other tasks during unassisted walking and RAGT.**

| **Classification of Gait vs Other Tasks** | | | | | | | | | | |
| --- | --- | --- | --- | --- | --- | --- | --- | --- | --- | --- |
| **Author,**  **Year (Country)** | **Participants**  **(n=x)** | **EEG Device & Connectivity** | **Number of Electrodes & EEG Sampling Rate** | **Additional Data Collected** | **Classification Task**  **(Walking Condition)** | **Artifact Management and Referencing**  **Methods** | **Filtering Applied** | **Features Used** | **Classifier Used**  **(Mode)** | **Performance** |
| ***Unassisted Walking*** | | | | | | | | | | |
| **Alyan,**  **2024 [46]**  (Germany) | Healthy  (n=35) | actiCAP    Wired | 30    500 Hz | Eye Blink data. | Walk vs rest.      (Free overground) | ICA  AMICA  Principal component analysis (PCA) | Band-pass (0.1–40 Hz) | Spatial features (ICA, PCA) | SVM  (Offline) | 69% |
| **Artoni,**  **2018* [44]**  (Switzerland) | Healthy  (n=1) | actiCAP    Wired | 64    2000Hz | EMG | Walk vs rest.    (Free overground) | ICA | High-pass low cutoff (2 Hz) | Frequency-domain features | LDA  (Offline) | 88.52% - 95.60% |
| **Bajpai,**  **2023 [108]**  (India) | PwPD  (n=8) | MOVE    Wireless | 28    1000Hz | Kinematics | Pre FOG vs normal walk.    (Free overground) | ICA | Band-pass broadband (0.5–100 Hz) | Learned features (CNN-based) | EEGFogNet  IMUFogNet  (Offline) | 86.2% - 92.1% |
| **Bhatlawande,**  **2023* [68]**  (India) | Healthy  (n=8) | Clarity Brain Tech device    Wired | 6    NR | N/A | Walk vs rest vs idle.    (Free overground) | NR | Band-pass broadband (10–500 Hz) + notch | Time–frequency features (spectrogram-based) | Random Forest (RF)  (Offline) | 100% |
| **Cho,**  **2023* [71]**  (USA) | PwPD  (n=12) | MOVE    Wireless | 25    1000Hz | EMG | FOG vs normal walk.    (Free overground) | ICA | Band-pass broadband (0.5–100 Hz) | Learned features (raw EEG) | Polynomial Regression    Dense Neural Network    Bayesian Neural Network    Autoencoder  (Offline) | **Polynomial Regression:** 61.1% - 64.6%    **Dense Neural Network:** 66%    **Bayesian Neural Network:** 55.6%    **Autoencoder:** 63% |
| **Choi,**  **2018* [69]**  (Korea) | Healthy  (n=5) | g.Nautilus    Wireless | NR    NR | EMG  Kinematics | Gait initiation vs standing.    Gait termination vs standing.    (Free overground) | ASR | NR | Spatial features; common spatial pattern (CSP) | LDA  (Offline) | **Initiation vs standing:**  20-60%    **Termination vs standing:**  20-60% |
| **Grana,**  **2020 [113]**  (Spain) | Healthy  (n=16) | Enobio    Wireless | 8    500Hz | Kinematics | Walk vs 9 other tasks.    (Free overground) | Automated artifact rejection during acquisition | Band-pass (0.1–50 Hz) | Frequency-domain features | RF  (Offline) | >90%. |
| **Handojoseno,**  **2012* [55]**  (Australia) | PwPD  (n=26) | Custom device    Wireless | 6    500Hz | NR | Normal walk vs pre-FOG.    Normal walk vs FOG.    (Free overground) | NR | Band-pass (0.5–60 Hz) + notch + noise removal | Time–frequency features (wavelet) | Three-layer back propagation neural network  (Offline) | **Walk vs pre-FOG:** 76.6%    **Walk vs FOG:** 73.4% |
| **Handojoseno,**  **2013* [56]**  (Australia) | PwPD  (n=26) | Custom device    Wireless | 6    500Hz | Video Recording | Pre FOG vs walk.    (Free overground) | Automated wavelet-based artifact reduction | Band-pass (0.5–60 Hz) + notch | Time–frequency features (wavelet) | Multilayer perceptron neural network (MLPNN)  (Offline) | 71.75% |
| **Handojoseno,**  **2014* [57]**  (Australia) | PwPD  (n=10) | Custom device    Wireless | 6    500Hz | NR | Transition to FOG vs FOG.    (Free overground) | NR | Band-pass (0.15–100 Hz / 0.5–60 Hz) + notch | Connectivity features | MLPNN  (Offline) | 78.04% |
| **Handojoseno,**  **2015 [58]**  (Australia) | PwPD  (n=16) | Custom device    Wireless | 6    500Hz | Video Recording | Pre FOG vs walk.    (Free overground) | Automated wavelet-based artifact reduction | Band-pass (0.15–100 / 0.5–60 Hz) + notch + wavelet | Hybrid features (entropy, spectral, connectivity) | MLPNN  (Offline) | **In Group (training and testing):** 80.2%    **Out Group (testing only):** 76.67% |
| **Handojoseno,**  **2018 [59]**  (Australia) | PwPD  (n=16) | Custom device    Wireless | 6    500Hz | Video Recording | Pre FOG vs walk.    (Free overground) | ICA | Band-pass (0.5–60 Hz) + notch | Connectivity features | Three-layer Back Propagation Neural Network  (Offline) | **Sensitivity**  In Group: 82.65%  Out Group: 85.86%    **Specificity**  In Group: 86.60%  Out Group: 80.25% |
| **Hasan,**  **2019* [73]**  (USA) | Healthy  (n=7) | Custom device    Wireless | 8    512Hz | EMG  Kinematics | Intention to start/intention to stop vs Walk/Relax.    (Free overground) | ICA  ASR | High-pass 1 Hz + notch | Time–frequency & time-domain features | SVM with Gaussian or Radial Basis Kernel  (Pseudo online) | **TP Rate**  70% minimum specificity required: 68.67%    90% minimum specificity required: 31.73% |
| **Hasan,**  **2021* [114]**  (USA) | Healthy  (n=6) | actiCAP    Wireless | 8    512Hz | EMG  Kinematics | Rest vs intention to walk.    Walk vs intention to stop.  (Free overground) | ICA  ASR | High-pass 0.1–1 Hz + notch | Time–frequency features (ERD) | SVM Radial Basis Kernel  (Offline) | **Rest vs Start:** 83.36%    **Walk vs stop:** 81.57% |
| **Hasan,**  **2021 [79]**  (USA) | Healthy  (n=1) | actiCAP    Wireless | 16    512Hz | Kinematics | Acceleration vs walking.    (Free overground) | ICA  AMICA  PCA  ASR | Band-pass (0.05–5 Hz) + notch (offline/pseudo online) | Time–frequency features (ERD, MRCP) | SVM Radial Basis Kernel  (Offline) | 85.9% |
| **Hortal,**  **2015* [115]**  (Spain) | Healthy  (n=3) | g.LADYbird  Wired    g.GAMMAcap  Wired | 32    1200Hz | Kinematics | Walk vs relax.  (Free overground) | NR | Band-pass (5–40 Hz) + notch + LP 100 Hz | Time–frequency features (ERD) | SVM Radial Base Function (RBF) Kernel  (Offline) | **Walk:** 75%  **Relax:** 70.5% |
| **Hortal,**  **2015* [116]**  (Spain) | PwSCI  (n=4) | g.LADYbird  Wired    g. GAMMAcap  Wired | 32    1200Hz | Kinematics | Intention to stop vs intention to start vs walk vs rest.    (Free overground) | NR | Band-pass (0.2–40 Hz) + notch + LP 100 Hz | Hybrid features (ERD, spectral) | SVM including a RBF  (Offline) | **Intention to start:**  51.5% - 85.2%    **Intention to stop:** 53.3%- 88.5%    **All classes:** 50%- 86.8% |
| **Hortal,**  **2016 [117]**  (Spain) | Healthy  (n=7)    PwSCI  (n=3) | g.GAMMAcap  Wired (Offline)    actiCAP  Wired (Online) | 9    1200Hz (Offline)    500Hz (Online) | Kinematics | Start walk vs walk vs stop walk vs stand.    (Free overground) | NR | Band-pass (Laplacian + 8th order 5–40 Hz) + notch | Time–frequency features (ERD/ERS) | SVM with RBF Kernel  (Offline/pseudo online, online) | **TP Rate**  *Offline/Pseudo Online*  50.6% - 56.4%    *Online*  Start: 56.3%  Stop: 58.3% |
| **Huang,**  **2019* [118]**  (China) | Healthy  (n=20) | EPOC    Wired | 14    128Hz | NR | Walking vs other (sitting & meditation).  (Free overground) | NR | NR | Frequency-domain features | Random Forest  (Offline) | 87.08% |
| **Hussain,**  **2023 [45]**  (USA) | Healthy  (n=75) | BioNomadix    Wireless | 6    2000Hz | NR | Walk vs rest vs reading vs other ‘working’ motor tasks.  (Free overground) | ICA  Rejection of segments  FastICA | Band-pass (0.5–44 Hz) + notch | Nonlinear & frequency-domain features | Random Forest with GBoost &  XGBoost  (Offline) | 80.33% |
| **Jochumsen,**  **2020 [119]**  (Denmark) | Healthy  (n=13) | g.GAMMAcap    Wired | 64    1200Hz | NR | Walk vs stand-to-sit vs sit to stand vs step up vs  sidestep vs back step.  (Free overground) | Rejection of segments. | Band-pass (0.1–30 Hz) | Hybrid features (time & frequency) | Random Forest with 512 trees  (Offline) | **Walking vs idle:** 83%    **All classes:** 54% |
| **John,**  **2023 [120]**  (Australia) | PwPD  (n=17) | Biosemi ActiveTwo    Wired | 32    500Hz | Video recording. | FOG onset vs walk vs initiation of voluntary stopping.    (Free overground) | NR | Band-pass (1–30 Hz) | Learned features (multichannel time series) | Deep ConvNet  (Offline) | **Transition to FOG vs transition to voluntary stop**: 92.28%    **Transition to FOG vs transition to voluntary stop vs normal walking**: 75.43% |
| **Li,**  **2024 [82]**  (China) | PwPD  (n=10) | MOVE    Wireless | 32    1000Hz | Video recording. | Normal walk vs transition to FOG vs FOG.      (Free overground) | ICA | Low-pass 62.5 Hz + notch 50 Hz | Spatial features (CSP) | CNN  Dynamic mode decomposition-adaptive common spatial pattern (DMD-ACSP)  (Offline) | **FOG Detection**  CNN: 89.2%  DMD-ACSP: 86.4%    **FOG Prediction**  CNN:84%  DMD-ACSP: 86.5% |
| **Lisi,**  **2015 [81]**  (Japan) | Healthy  (n=8) | Biosemi ActiveTwo    Wired | 64    2048Hz | Kinematics  Video recording. | Normal walk vs gait speed changes.    (Treadmill) | ICA-based artifact rejection with automated independent component classification | Band-pass (1–45 Hz / 8–40 Hz) | Time–frequency features (PCA-reduced) | Sparse Logistic Regression  (Offline, pseudo online) | **Offline:** 58% - 81%    **Pseudo Online:**  0.6 - 0.89 (area under curve) |
| **Long,**  **2018* [75]**  (China) | Healthy  (n=4) | Biosemi ActiveTwo    Wired | 32    512Hz | Kinematics | Intention to step over obstacles vs walk.  (Free overground) | NR | Multi-band-pass (0.3–30 Hz windows) | Spatial features (CSP) | SVM  (Pseudo online) | 93.14% |
| **Lu,**  **2025 [29]**  (China) | Healthy  (n=12) | EPOC Flex    Wireless | 32    128Hz | Footswitch data. | Walk vs run vs stair descent vs stair ascent vs stand-to-sit vs sit to stand vs jump.    (Free overground) | Rejection of Segments.    CAR | Band-pass (1–30 Hz) | Spatial–time-domain features | CSP Graph attention network  (Offline) | **Precision**  Walk: 97.22%  Run: 92.68%    **Sensitivity**  Walk: 95.93%  Run: 93.89%    **Specificity**  Walk: 99.36%  Run: 99.68% |
| **Ly,**  **2017* [83]**  (Australia) | PwPD  (n=5) | Biosemi ActiveTwo    Wired | 15    512Hz | Video recording. | Walk vs gait initiation failure.  (Free overground) | AAR (Automatic Artefact Removal) | Band-pass (0.5–50 Hz) | Hybrid features (PCA, ICA, wavelet) | SVM with RBF  (Offline) | 86.3% |
| **Mesin,**  **2022 [121]**  (Italy) | PwPD  (n=12) | MOVE    Wireless | 28    500Hz | EMG  Kinematics  Video recording. | FOG vs non-FOG walk.  (Free overground) | Automatic removal artifact using toolbox. | Band-pass (1.6–30 Hz) | Hybrid features (entropy, power spectral density, coherence) | SVM    K-nearest neighbours (KNN)  (Offline) | **KNN:** 87.65%  **SVM:** 88% |
| **Murugavalli,**  **2022 [70]**  (India) | Healthy  (n=24) | NR    NR | 32    NR | NR | Stand vs slow walk vs fast walk vs running.  (Treadmill) | NR | Band-pass (low/high) | Time–frequency features (wavelet) | Artificial neural network.    LSTMRNN  (Offline) | **ANN:** 69.35%    **LSTMRNN:** 96.7% |
| **Nidhi,**  **2021* [61]**  (India) | Healthy  (n=10) | actiCAP    Wireless | 64    100Hz | EMG  Kinematics | Walking vs ramp ascent vs ramp descent vs standing vs stairs ascent vs stairs descent.  (Free overground) | ASR  CAR  RELiable ICA (RELICA)  Electrooculography channels removed | High-pass 0.1 Hz | Spatial features (PCA, ICA) | Custom 1D CNN  (Offline) | 68% |
| **Ortiz,**  **2017* [122]**  (Spain) | Healthy  (n=8) | actiCAP    Wireless | 32    500Hz | Kinematics | Walk start and walk stop.  (Free overground) | CAR | Band-pass (0.2–100 Hz) + notch + spatial | Time–frequency features (ERD/ERS) | SVM Radial Basis Kernel  (Offline) | **Start:** 79.26%  **Stop:** 88.1% |
| **Ortiz,**  **2017 [123]**  (Spain) | Healthy  (n=8)    PwSCI  (n=8) | actiCAP    Wired | 31    500Hz | Kinematics | Walk start/walk stop vs relax/walk.  (Free overground) | NR | Band-pass (0.2–100 Hz) + notch + Laplacian | Frequency-domain features. | SVM  (Offline) | **Start:** 83.48%  **Stop:** 88.97% |
| **Park,**  **2019* [124]**  (Korea) | Healthy  (n=3)    Stroke Survivors (chronic)  (n=3)    Stroke Survivors (sub-acute)  (n=8) | g.Nautilus    Wireless | 32    500Hz | NR | Walk vs stand.    Walk vs stand ready.    (Free overground) | ASR | Band-pass (3–40 Hz) + band-stop (59–61 Hz) | Hybrid features (temporal, spectral, spatial) | CNN  (Offline) | ***Walk vs Stand***  Healthy: 77.4%  Stroke survivors (sub-acute): 85.9%  Stroke survivors (chronic): 85.2%      ***Walk vs Intention to Stand***  Healthy: 81.8%  Stroke survivors (sub-acute): 71.3%  Stroke survivors (chronic): 84.3% |
| **Preethi**  **2023* [72]**  (India) | PwPD  (n=12) | MOVE    Wireless | 32    1000Hz | EMG  Kinematics | FOG vs non-FOG.  (Free overground) | Removal of noisy/  missing data. | Filtered for noise/  missing values | Learned features | CNN-BiLSTM with attention  (Offline) | 95.8% |
| **Quiles,**  **2022 [74]**  (Spain) | Healthy  (n=8) | actiCHamp bundle    Wireless | 27    500Hz | Kinematics | Normal walk vs turning intention.    (Free overground) | ASR | High-pass 0.1 Hz + notch + H∞ filter | Connectivity features (covariance matrices) | SVM    Minimum Distance to Mean (MDM)    MDM with filter    LDA  (Offline, pseudo online, online) | **Offline**  SVM: 60.6%  MDM: 63.1%  MDM filter: 62.6%  LDA: 62.1%    Generic electrodes:  65.1–66.2%    Personalized electrodes:  68.2–69.6%    **Pseudo Online**  Generic electrodes:  TP: 41.4%  False positives (FP)/min: 9.4    Personalised electrodes:  TP: 40.2%  FP/min: 9.3    **Online**  SVM: 77.7% |
| **Severens,**  **2015 [76]**  (Netherlands) | Healthy  (n= 18)    9 Offline    12 Online | TMSi Refa-72    Wired | 62    500Hz | NR | Walk vs not walking.    (Treadmill) | CAF    Canonical correlation analysis (CCA)    Component Analysis | Laplacian filter | Frequency-domain features | Regularized Logistic Regression  (Offline, online) | **Offline**  Forward Walk: 78%  Backward Walk: 82%    **Online**  Constant speed walk: 78%  Adaptive speed walk: 70% |
| **Shafiul Hasan,**  **2020 [125]**  (USA) | Healthy  (n=6) | actiCAP    Wired | 8    500Hz | EMG  GRF | Rest vs start.  Walk vs stop.    (Free overground) | ICA  ASR  AMICA | High-pass 0.1 Hz + notch | Time–frequency features (ERD) | SVM RBF kernel (Offline) | **Rest vs start:** 88.23%    **Walk vs stop:** 87.04% |
| **Soangra,**  **2023 [126]**  (Spain) | Healthy  (n=13) | actiCAP    Wired | 64    500Hz | Kinematics | Walk vs walk unstable i.e. perturbations vs gait with mental task vs gait with feedback.  (Treadmill) | ICA | Band-pass (1–70 Hz) + notch 55–65 Hz | Hybrid features; entropy, power spectral density, wavelet. | RNN    Chrono Net  (Offline) | **Chrono Net**  4 Classes: 68%  3 Classes: 77.4%    **RNN**  2 classes: 82% |
| **Soriano-**  **Segura,**  **2021 [80]**  (Spain) | Healthy  (n=6) | G.tec device    Wired | 32    1200Hz | Kinematics | Walk vs intention to change direction while walking.  (Free overground) | Rejection of Segments | Band-pass (0.5–40 / 8–13 Hz) + notch + Laplacian | Hybrid features (frequency & temporal) | KNN  (Offline) | **Sensitivity**  Walk: 93.3%    Change direction: 93.9% |
| **Tortora,**  **2020* [77]**  (Italy) | Healthy  (n=12) | actiCAP    Wired | 64    2048Hz | EMG | Walk vs stand.    (Treadmill)    (Free overground) | ICA  ASR  CAR  RELICA  Automatic rejection. | Band-pass (1–98 Hz) + notch | Nonlinear features (entropy) | LDA  (Offline, online) | **Offline:** 89%    **Online:** 85.6% |
| **Yu,**  **2025 [62]**  (China) | Healthy  (n=3) | Neuro  Workbench    Wired | 16    500Hz | Kinematics | Normal walk vs anticipated fall.    (Free overground) | ICA    Rejection of Segments    Blind source separation | Band-pass (0–40 Hz) / (0.1–40 Hz) / (0.5–40 Hz) | Nonlinear features (entropy) | SVM    Random Forest    KNN  (Offline) | **RF:** 85% |
| **Zafar,**  **2024 [127]**  (Italy) | Healthy  (n=10) | EPOC Flex    Wireless | 14    256Hz | NR | Ascending stairs vs descending stairs vs walking ramps vs walk.  (Free overground) | ICA    Adjust ICA | Band-pass (unspecified) | Hybrid features (time & frequency) | RF    KNN  (Offline) | **RF:** 93%  **KNN:** 80% |
| **Zhang,**  **2016* [128]**  (China) | Healthy  (n=4) | NR    NR | 25    256Hz | NR | Walking vs run vs stand.  (Free overground) | CAR. | Band-pass (0.5–45 Hz) | Spatial features (CSP, ICA) | Small World Neural Network  (Offline) | 88.29% |
| ***RAGT*** | | | | | | | | | | |
| **Choi,**  **2019* [66]**  (Korea) | Healthy  (n=3) | actiCAP Twist    Wireless | 31    500Hz | EMG | Walk vs stand.    (Exoskeleton) | ASR | Band-pass (3–40 Hz) + band-stop (59–61 Hz) | Spatial features (CSP) | LDA  (Offline, online) | **Offline**  *Stand vs gait*  Min: 70%  Max: 82.6%    **Online**  *Stand vs start/stop*  Start: 92.3%  Stop: 100% |
| **Duan,**  **2022 [63]**  (China) | Healthy  (n=30) | ASA-Lab    Wired | 62    1000Hz | EMG | Walk vs exoskeleton free walk vs exoskeleton low assist vs exoskeleton high assist.    (Free overground)    (Exoskeleton) | ICA | Band-pass (0.5–45 Hz) + adaptive electrooculography (EOG) removal | Time–frequency features (wavelet) | Multi-Scale Learning  (Offline) | **Group level:**  20 channel EEG: 60.34%    62 channel EEG: 74.26%    20 channel EEG & EMG: 88.82%    62 channel EEG & EMG: 89.33%    **Individual level:**  High assist: 85.92%    Low assist: 83.59%    Zero assist: 90.85%    Free walk: 98.24% |
| **Garcia-**  **Cossio,**  **2015 [37]**  (Netherlands) | Healthy  (n=10)    Stroke Survivors  (n=3) | TMSi Refa- 72    Wired | 62    500Hz | EMG  Kinematics | Active walk vs passive walk.    Also walk vs standing.    Passive walk vs standing.    (RAGT treadmill) | CCA | Band-pass (8–40 Hz) + detrending | Time–frequency features (power spectral density, ERD/ERS) | L2-regularized logistic regression classifier  (Offline) | **Healthy**  *Active RAGT vs stand:* 94%    Active RAGT vs passive RAGT: 83.4%    **Stroke Survivors**  Walking vs baseline: 89.8% |
| **Goh,**  **2018 [78]**  (Singapore) | Healthy  (n=27) | ASA-Lab    Wired | 62    1000Hz | NR | Free walk vs exoskeleton zero assist vs exoskeleton low assist vs exoskeleton high assist.    (Free overground)    (Unilateral Exoskeleton) | ICA  CCA | Band-pass (0.5–45 Hz) + least mean squares filtering | Spatial & frequency-domain features | Spatio-spectral representation learning (SSRL)    RF SVM with RBF Kernel.  (Offline) | **SSRL:** 77.8% |
| **Hehenberger,**  **2017* [60]**  (Austria) | Healthy  (n=12) | Easycap  NR  (Offline)    Eego sports  NR  (Online) | 120  (Offline)  2.5kHz    64  (Online)  500Hz | EMG  Ground contact forces. | Walking vs standing.    (RAGT treadmill)    (Treadmill) | CAR | Band-pass 1–200 Hz + notch (offline); High pass 1 Hz (online) | Time–frequency features (ERD, wavelet) | LDA  (Offline, online) | **Offline**  RAGT Treadmill:  78% - 98%    **Online**  Treadmill:  80.88% - 92.56% |
| **Jeong,**  **2020 [65]**  (Korea) | Healthy  (n=10) | actiCAP    Wireless | 32    1000Hz | EMG | Walk intention vs rest.  (Free overground)    (Exoskeleton) | NR | Band-pass (0.05–10 Hz) + notch + 30-band filter bank | Time-domain features (MRCP) | Regularized LDA  (Offline, pseudo online) | **Offline:** 86%    **Pseudo Online:** 86% |
| **Quiles,**  **2022 [64]**  (Spain) | Healthy  (n=5) | actiCHamp    Wired | 32    500Hz | Kinematics  EOG | Normal walk vs intention to change speed of gait.    (Free overground)    (Exoskeleton) | Adaptive artifact EOG removal algorithm H∞. | Band-pass + high-pass 0.1 Hz + notch + Laplacian | Hybrid features (entropy, power spectral density, nonlinear) | Binary SVM with RBF kernel.  (Offline, pseudo online, online) | **Offline**  66.1%    **Pseudo Online**  TP: 42%  FP: 9 per min    **Online** (Exoskeleton only)  FP/min: 9.92  TP: 33% |
| **Quiles,**  **2023 [67]**  (Spain) | Healthy  (n=8)    PwSCI  (n=2) | actiCAP    Wired | 27    500Hz | Kinematics | Walk vs stopping at an obstacle.    (Exoskeleton overground)    (Treadmill) | NR | Band-pass + high-pass 0.1 Hz + notch + ocular artifact removal | Learned features (raw EEG to CNNs) | Neural network 1 (NN1)    Neural network 2 (NN2)  (Pseudo online, online.) | **Healthy**  *Pseudo-Online Treadmill*  NN1: 89.3% (TP/R)  NN2: 75.8% (TP/R)    *Pseudo Online Exoskeleton*  NN1 & NN2: 74.2% (TP/R)    *Online Exoskeleton*  TP/R: 91.7%.  FP/min: 3.8.    **PwSCI**  *Online Exoskeleton*  TP/R:100%  FP/min: 7.7. |
| **Zhang,**  **2017 [31]**  (USA) | Healthy  (n=1)    PwSCI (n=1) | actiCAP    Wireless | 64    100Hz | NR | Walk vs Stop.    (Exoskeleton overground) | ASR | Band-pass (0.1–2 Hz) | Spatial features (Region of interest amplitude modulation) | Multiple Kernel Learning  (Offline) | 65% |

**Footnotes:** * denotes conference paper**.** Filtering information is summarized. Exact cutoff frequencies and filter parameters vary across studies and are reported in the original publications. Feature descriptions are grouped into broad methodological categories (e.g., time-domain, frequency-domain, time–frequency, spatial, connectivity, nonlinear, and learned features) to improve clarity and comparability across studies. Performance is reported as percentage accuracy unless otherwise stated. TP denotes true positive detection rate (% of gait events detected within the predefined temporal tolerance). N/A indicates metric not applicable. NR indicates data was not reported.

**Appendix 3: PRISMA-ScR (Preferred Reporting Items for Systematic reviews and Meta-Analyses extension for Scoping Reviews) Checklist.**

| **SECTION** | **ITEM** | **PRISMA-ScR CHECKLIST ITEM** | **REPORTED** |
| --- | --- | --- | --- |
| **TITLE** | | | |
| Title | 1 | Identify the report as a scoping review. |  |
| **ABSTRACT** | | | |
| Structured summary | 2 | Provide a structured summary that includes (as applicable): background, objectives, eligibility criteria, sources of evidence, charting methods, results, and conclusions that relate to the review questions and objectives. |  |
| **INTRODUCTION** | | | |
| Rationale | 3 | Describe the rationale for the review in the context of what is already known. Explain why the review questions/objectives lend themselves to a scoping review approach. |  |
| Objectives | 4 | Provide an explicit statement of the questions and objectives being addressed with reference to their key elements (e.g., population or participants, concepts, and context) or other relevant key elements used to conceptualize the review questions and/or objectives. |  |
| **METHODS** | | | |
| Protocol and registration | 5 | Indicate whether a review protocol exists; state if and where it can be accessed (e.g., a Web address); and if available, provide registration information, including the registration number. |  |
| Eligibility criteria | 6 | Specify characteristics of the sources of evidence used as eligibility criteria (e.g., years considered, language, and publication status) and provide a rationale. |  |
| Information sources* | 7 | Describe all information sources in the search (e.g., databases with dates of coverage and contact with authors to identify additional sources), as well as the date the most recent search was executed. |  |
| Search | 8 | Present the full electronic search strategy for at least 1 database, including any limits used, such that it could be repeated. |  |
| Selection of sources of evidence† | 9 | State the process for selecting sources of evidence (i.e., screening and eligibility) included in the scoping review. |  |
| Data charting process‡ | 10 | Describe the methods of charting data from the included sources of evidence (e.g., calibrated forms or forms that have been tested by the team before their use, and whether data charting was done independently or in duplicate) and any processes for obtaining and confirming data from investigators. |  |
| Data items | 11 | List and define all variables for which data were sought and any assumptions and simplifications made. |  |
| Critical appraisal of individual sources of evidence§ | 12 | If done, provide a rationale for conducting a critical appraisal of included sources of evidence; describe the methods used and how this information was used in any data synthesis (if appropriate). | N/A |
| Synthesis of results | 13 | Describe the methods of handling and summarizing the data that were charted. |  |
| **RESULTS** | | | |
| Selection of sources of evidence | 14 | Give numbers of sources of evidence screened, assessed for eligibility, and included in the review, with reasons for exclusions at each stage, ideally using a flow diagram. |  |
| Characteristics of sources of evidence | 15 | For each source of evidence, present characteristics for which data were charted and provide the citations. |  |
| Critical appraisal within sources of evidence | 16 | If done, present data on critical appraisal of included sources of evidence (see item 12). | N/A |
| Results of individual sources of evidence | 17 | For each included source of evidence, present the relevant data that were charted that relate to the review questions and objectives. |  |
| Synthesis of results | 18 | Summarize and/or present the charting results as they relate to the review questions and objectives. |  |
| **DISCUSSION** | | | |
| Summary of evidence | 19 | Summarize the main results (including an overview of concepts, themes, and types of evidence available), link to the review questions and objectives, and consider the relevance to key groups. |  |
| Limitations | 20 | Discuss the limitations of the scoping review process. |  |
| Conclusions | 21 | Provide a general interpretation of the results with respect to the review questions and objectives, as well as potential implications and/or next steps. |  |
| **FUNDING** | | | |
| Funding | 22 | Describe sources of funding for the included sources of evidence, as well as sources of funding for the scoping review. Describe the role of the funders of the scoping review. |  |

**Appendix 4: Search string.**

EEG OR electroencephalog* OR "electro encephalogram*"

*AND*

Walk* OR step* OR gait OR locomot* OR ambulat* OR “heel strike” OR “toe off” OR stance OR swing OR stride*

*AND*

Classif* OR coupl* OR recognition OR recognise OR recognize OR categorisation OR categorization OR categorise OR categorize OR analy* OR identif* OR correlat* OR decod* OR characteri*

**Note:** *Asterisks (*) denote truncation symbols, whereby the search retrieves all variations of a word stem. For example, "classif*" would retrieve classify, classification, classifier, classifying etc.*
